# Peritraumatic distress associated with domestic violence

**DOI:** 10.1192/j.eurpsy.2023.2404

**Published:** 2023-07-19

**Authors:** R. Jbir, L. Aribi, N. Mseddi, I. Chaari, F. Charfeddine, S. Ellouze, J. Aloulou

**Affiliations:** Hedi Chaker university hospital, Sfax, Tunisia

## Abstract

**Introduction:**

The violence against women massively committed by their spouses is a scourge that transcends countries, ethnicities, cultures, classes social and age groups.

This violence is traumatic and represents a serious attack on the physical integrity and mental health of the women who are victims

**Objectives:**

To study the prevalence and predictors of peritraumatic distress among women victims of domestic violence

**Methods:**

We contacted 122 women who consulted at the psychiatric emergency of ‘Hedi Chaker hospital’,Sfax examined in the context of medical expertise on the period between May 2021 until January 2022

A questionnaire regarding the violence was asked to responders .It included demographic and violence exposure questions and a scale applied during violence ‘Peritraumatic distress inventory’

**Results:**

The average age of women assaulted in our study was 35.6 ± 9.94 years (min=18,max=64).

78.7% (n=96) of ladies were of urban origin.

The majority of them(44,3%) had secondary level education.

The half of the population (51.6%) had an average socio-economic level. (86.1%) had children.

98.7%were victim of verbal violence,94.7% of physical violence, 97.3% of psychological violence and 54.7 %of sexual violence.

72.1% of women (N=88) developed peritraumatic distress related to the assault with a risk of developing post-traumatic stress disorder.

Women who were threatened by their spouses were more in distress than others (0,04).

Physically abused women using a knife developed more peritraumatic distress (p=0,02).

**Conclusions:**

Domestic violence is a global public health problem, that calls for urgent actions.Peritraumatic distress linked to violence may lead to psychotraumatic disordersthat are the source for traumatized victims of great suffering mental health and a possible vital risk (suicide, risky behavior).

**Disclosure of Interest:**

None Declared

